# Medical Students’ General Beliefs and Specific Perceptions about Patient Feedback Before and after Training in a Clinical Context

**DOI:** 10.5334/pme.1261

**Published:** 2024-05-08

**Authors:** M. Charlotte L. Eijkelboom, Renske A. M. de Kleijn, Joost Frenkel, Marieke F. van der Schaaf

**Affiliations:** 1 Utrecht University, the Netherlands; 2 Department of Pediatrics, University Medical Center Utrecht, the Netherlands; 3 Utrecht Center for Research and Development of Health Professions Education, University Medical Center Utrecht, the Netherlands

## Abstract

**Introduction:**

Despite its high potential, patient feedback does not always result in learning. For feedback to be effective students must engage with it, which partly depends on their perceptions of feedback. To better understand student engagement with patient feedback in a clinical context, this study explored the following research questions: 1) What are medical students’ general beliefs about patient feedback and what are their specific perceptions of feedback messages? 2) What is the difference between these general beliefs and feedback message perceptions before and after patient feedback training?

**Methods:**

The study context was a 12-week clerkship combining Pediatrics and Gynecology, which included feedback training for students and asking for patient feedback. Ninety 4^th^-year medical students completed pre- and post-clerkship questionnaires. The questionnaires (Beliefs about Patient Feedback Questionnaire, Feedback Perception Questionnaire) were adapted from validated peer-feedback questionnaires. Questionnaires were quantitatively analyzed.

**Results:**

Both pre- and post-clerkship, students had positive general beliefs about patient feedback and positive perceptions of the feedback messages they received. However, paired t-tests showed that students’ general beliefs and feedback message perceptions became less positive after feedback training and experience.

**Discussion:**

Patient feedback is not an easy means to learn and students do not become feedback literate in terms of patient feedback overnight. We suggest that future researchers further explore reasons for the decline in positive perceptions of patient feedback. We suggest implementing longitudinal feedback training in medical curricula, where students are guided and supported in the complex task of learning from patients through feedback.

## Introduction

Feedback is an important driver of learning in medical education [1]. Patient feedback is increasingly considered an important means to learn, since it could provide an additional perspective to that of healthcare professionals [2,3]. Yet, despite its high potential, patient feedback does not always result in learning. Reviews on the effects of patient feedback on medical performance show mixed results in terms of self-reported feedback use, self-reported intention to change, and measured change in medical performance [3,4,5]. Possible explanations for these mixed results are differences in perceptions about patients as feedback providers and feedback messages, as was seen in the review study of Baines *et al*.: positive perceptions about patients as feedback providers and feedback messages led to more acceptance and (self-reported) use of patient feedback [4].

To further understand patient feedback, this study focuses on students as users of patient feedback. A recent paradigm shift in feedback literature caused for feedback to no longer be seen as information which should be well delivered by a feedback provider. Instead, feedback is now seen as a co-constructive process, in which both the provider and learner have an active role [6,7]. For feedback to be effective learners must engage with feedback, by seeking, interpreting and acting upon it [8]. Feedback engagement partly depends on learners’ general beliefs and specific perceptions about feedback interactions. General beliefs about the value of feedback and one’s own role in the feedback process, specific perceptions about a feedback interaction, a feedback provider, and a feedback message all impact feedback engagement [8,9,10,11,12,13,14].

### General beliefs about patient feedback

General beliefs are considered to be precursors to attitudes and to guide behavior [15]. Carless and Boud argue that recognizing the value of feedback and understanding one’s own role in the process is a feature of feedback literacy and promotes acting upon feedback [16]. Studies about peer- or supervisor-feedback report that students’ beliefs about the purpose and value of feedback, influence their feedback seeking behavior and feedback use [8,9,10,11,12]. In the context of peer-feedback, Huisman *et al*. argued that beliefs about feedback comprise multiple aspects, namely valuation of feedback as an instructional method, confidence in the quality of feedback messages, and valuation of feedback skills [17,18].

With regard to general beliefs about patient feedback, for doctors both positive [19,20,21,22,23] and negative aspects [20,23,24,25] have been reported. Positive aspects regarded valuation of patient feedback as a self-directed learning method to improve clinical skills [20,23] and patients being consumers of healthcare and therefore provide unique and important feedback [19,22]. Negative aspects regarded concerns about the quality of feedback messages, since patients might not have the knowledge to provide feedback [24], provide feedback on matters which are not directly related to the doctor’s performance [20], are unreasonably critical, or providing socially acceptable answers [23]. For students, positive general beliefs about patient feedback have been reported [26,27,28]. Gran *et al*. found that medical students were interested in patients’ feedback on their consultation skills during their general practice clerkship [27]. Yet, student social care support workers expressed concerns about the quality of the feedback message, since they questioned whether patients would give honest feedback due to perceived unequal power balance [29]. In sum, research has been done on separate aspects of students’ general beliefs about patient feedback, namely valuation of patient feedback as an instructional method and confidence in quality of the messages. Yet, little is known about students’ beliefs of their own feedback skills in the context of patient feedback. Moreover, little is known about how the various aspects of feedback beliefs combine. Moreover, a validated comprehensive measure of students’ beliefs about patient feedback is missing, which makes it hard to follow-up and align research findings.

### Feedback message perceptions

To act upon a feedback message, learners must first perceive the feedback message [30]. Strijbos *et al*. defined feedback message perceptions as the outcomes of how learners experience the feedback message content. These perceptions can relate to the cognitive function of feedback, such as perceptions regarding the fairness, usefulness and acceptability of the feedback message content. Or they can relate to the motivational function of feedback and whether it motivates to improve performance [31]. Perceptions of feedback messages mediate the relation between the received feedback message and subsequent performance [31].

With regard to perceptions of patient feedback, doctors and students report receiving non-specific and mainly positive feedback from patients [22,28,32]. For doctors, studies showed that whether they accept and use patient feedback is influenced by their perceptions on the specificity of the feedback message, whether it is positive or constructive, and whether the message is congruent with their self-perceptions [4,22,25]. For medical students, Chua *et al*. reported that they considered positive general feedback from patients as unhelpful, whereas Gran *et al*. found that positive feedback was regarded as either encouraging or untrustworthy [27,28]. Still, for students it is unknown how these perceptions affect their intention to act upon the message.

### Training in patient feedback

As argued above, feedback engagement is influenced by general beliefs about feedback and specific perceptions regarding feedback messages. Yet, engagement with feedback can also impact general beliefs and feedback message perceptions [11,22]. In their review on peer-feedback in higher and primary education, van Zundert *et al*. showed that student general beliefs regarding formative and summative peer assessment were positively influenced by training and experience [33]. With regard to feedback message perceptions, Bogetz *et al*. showed that discussing patient feedback with a faculty member in a facilitated reflection session, facilitated recall of patient encounters, which helped residents to understand their feedback and develop specific learning goals [22]. Feedback training and experience seems to positively influence feedback beliefs and feedback message perceptions. Yet, it is unknown whether this holds true for medical students engaging with patient feedback.

In sum, to better understand student engagement with patient feedback, this study explores medical students’ general beliefs about patient feedback and their specific perceptions of the feedback messages they received from patients. We explore whether these general beliefs and feedback message perceptions change after a patient feedback training, and whether these can be measured by adjusting validated questionnaires from peer-feedback literature.

The research questions of this study are:

1. What are medical students’ general beliefs about patient feedback and students’ specific perceptions of patient feedback messages?
2. What is the difference between students’ general beliefs about patient feedback and students’ feedback message perceptions before and after patient feedback training in a clinical clerkship?

## Method

### Study design

We used an explorative study design, where we collected quantitative questionnaire data pre- and post-patient feedback training.

### Context

**Figure 1:**
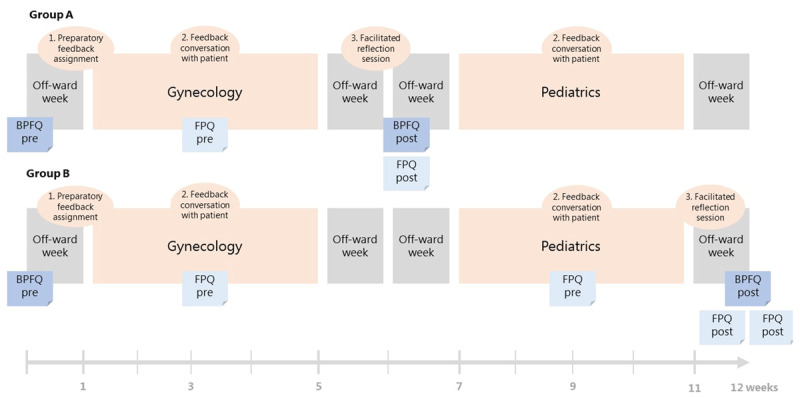
Study context and data collection. The study context was a 12-week clerkship consisting of 4 weeks gynecology, 4 weeks pediatrics and 4 off-ward weeks. The feedback training consisted of (1) a preparatory feedback assignment, (2) feedback conversations with patients and (3) a facilitated reflection session. Group A participated in the facilitated reflection session in week 5, group B participated in week 12. The questionnaires students completed during their clerkship are displayed in blue. BPFQ = beliefs about patient feedback questionnaire, FPQ = feedback perception questionnaire.

The study context is a 12-week clerkship in the Netherlands, consisting of 4 weeks Gynecology, 4 weeks Pediatrics and 4 off-ward weeks. (See Figure 1). During this clerkship students received feedback training and asked patients for feedback on their clinical performance. Feedback training consisted of (1) a preparatory feedback assignment, (2) feedback conversations with patients and (3) a facilitated reflection session. The training was developed using the Westerveld framework for feedback dialogues [34]. This framework centers around seven criteria: Open and respectful; Relevant; Timely; Dialogical; Responsive; Sense making; and Actionable. The preparatory feedback assignment entailed watching an instructive online video about asking patients for feedback, and setting individual learning goals on which students wanted to collect feedback. During their clerkship students had feedback conversations with at least one gynecology patient and one pediatric patient (or caregiver of a young patient). Feedback conversations were student initiated and face-to-face. Students documented their received feedback messages on an online educational platform. During a facilitated reflection session, students reflected on their feedback conversations using the Westerveld criteria for feedback dialogues, analyzed their feedback by making comparisons, and planned how they were going to implement what they learned from this feedback (See Appendix 1 for the lesson plan of the facilitated reflection session) [34,35]. These sessions were held in groups of 10–15 students and lasted 1.5 hours. Due to organizational reasons, half the students participated in the facilitated reflection session halfway through their clerkship, the other students participated at the end of their clerkship.

### Participants

We included fourth-year medical students from a Dutch University, who started their clerkship between March 2022 and July 2022 and completed their clerkship by September 2022. If a student did not participate in the facilitated reflection session they were excluded. Ethical approval of this study was provided by the Dutch Association for Medical Education (NVMO, NERB file number: 2021.8.8). All data was anonymized after data collection.

### Instruments

We reviewed the literature for validated and concise (to increase the response rate) questionnaires regarding general beliefs about feedback and regarding perceptions of a specific feedback interaction [17,18,31,36,37,38]. Regarding general feedback beliefs, the Beliefs about Peer Feedback Questionnaire (BPFQ) and the Feedback Orientation Scale met our criteria [17,36]. We chose to use the BPFQ, since this questionnaire could more easily be adjusted to the context of patient feedback. Regarding perceptions of a specific feedback interaction, the Feedback Perception Questionnaire (FPQ) and the FEEDME-provider questionnaire met our criteria [31,37]. We chose to use the FPQ since this questionnaire was validated in multiple contexts and contains a scale measuring intentions.

#### General beliefs about patient feedback

Students’ general beliefs about patient feedback were measured with an adapted version of the Beliefs about Peer Feedback Questionnaire [17]. The questionnaire consists of 4 scales, namely Valuation of peer-feedback as an instructional method (3 items), Confidence in own peer-feedback quality (2 items), Confidence in quality of received peer-feedback (2 items), and Valuation of peer-feedback as an important skill (3 items). We adjusted the questionnaire to fit the context of student-patient feedback interactions by removing the ‘Confidence in own peer-feedback quality’ scale, removing the first item of the “Valuation of peer-feedback as an important skill” scale, and replacing the words ‘peer’ and ‘student’ for the word ‘patient’. We tested the adjusted questionnaire in a focus group with 6 medical students, after which no adjustments deemed necessary. See Table 1 for the final questionnaire. Items were measured on a 5-point Likert scale, the labels ranged from 1 (completely disagree, or completely not applicable to me) to 5 (completely agree, or completely applicable to me).

**Table 1:** Beliefs about patient feedback questionnaire.


1.	Involving patients in my learning process through the use of patient-feedback is meaningful

2.	Patient-feedback within ‘this’ clerkship is useful

3.	Feedback should only be provided by clinical supervisors

4.	In general, I am confident that the feedback I receive from patients is of good quality

5.	In general, I am confident that the feedback I receive from patients helps me to improve my performance

6.	Being capable of dealing with critical patient-feedback is an important skill

7.	Being capable of improving one’s work based on received patient-feedback is an important skill


This questionnaire was developed by building on the Beliefs about peer-feedback questionnaire [17].

Since the questionnaire is relatively new and we used an adapted version in a new context, we performed an exploratory factor analysis to empirically explore the underlying structure of the questionnaire. We conducted principal component analysis with oblique (oblimin) rotation on the data of the first moment students filled in this questionnaire (n = 85). The pattern matrix and scree plot were used to determine the number of components. Analysis showed that all items of the questionnaire loaded on one component, which explained 44,78% of the total variance. Factor loadings of the items ranged from 0.51–0.81, see Appendix 2. Therefore, we chose to perform further analysis with one overarching scale, which we named the Beliefs about patient feedback scale (BPF). The internal consistency of this scale at moment 1 and 2 was sufficient (Cronbach’s alpha .80 and .83).

#### Perceptions of patient feedback messages

Students’ perceptions of the feedback messages from patients were measured with an adapted version of the Feedback Perceptions Questionnaire (FPQ) [31]. This questionnaire was chosen because it measures perceptions of a specific feedback message, on a specific task, by a specific source and showed good structural validity [31]. The questionnaire measures feedback message perceptions using three scales: Perceived adequacy of feedback (9 items), Willingness to improve (3 items) and Affect (6 items). We used 2 out of the 3 scales, namely the Perceived adequacy of feedback (FP_PAF_) and Willingness to improve (FP_WI_), to make the questionnaire more concise and thereby increase the response rate. The Perceived adequacy of feedback comprises items about the perceived fairness, usefulness, and acceptance of the feedback, and relates to the cognitive function of feedback. The Willingness to improve scale relates to the motivational function of feedback. We adjusted the second and third item of this scale to fit the context of face-to-face feedback conversations between medical students and patients in a hospital setting. After testing the adjusted questionnaire in a focus group with 6 medical students, minor adjustments regarding the phrasing of the Willingness to improve items were made. (See Table 2 for the final questionnaire.) Items were measured on a visual analogue scale from 0 (fully disagree) to 10 (fully agree).

**Table 2:** Feedback perception questionnaire (in context of patient feedback).


SCALE	ITEM

PERCEIVED ADEQUACY OF FEEDBACK	

1.	I am satisfied with this feedback

2.	I consider this feedback fair

3.	I consider this feedback justified

4.	I consider this feedback useful

5.	I consider this feedback helpful

6.	This feedback provides me a lot of support

7.	I accept this feedback

8.	I dispute this feedback

9.	I reject this feedback

**Willingness to improve**	

10.	Based on this feedback, I am willing to improve my performance

11.	I am willing to invest a lot of effort in improving the skills I received feedback on

12.	I am willing to, during my clerkships, work on further developing the skills I received feedback on


This questionnaire was developed by building on the Feedback Perception Questionnaire for peer-feedback. [31] All items answered on a bi-polar scale from 0 (fully disagree) to 10 (fully agree).

We used an adjusted version of the questionnaire in a new context, therefore we explored the underlying questionnaire structure by conducting factor analyses on the questionnaire data of the first moment students filled in this questionnaire. (See Appendix 3 for the complete analysis process.) Eventually, principal component analysis, with the factor amount fixed on 2, resulted in two components that explained for 57.06% of the total variance. The components matched the original questionnaire structure: component 1 consisted of the Perceived adequacy of feedback items; component 2 consisted of the Willingness to improve items. Factor loadings for the two scales ranged from .46–.91, see Appendix 3. The internal consistencies of both scales (FP_PAF_ and FP_WI_) was sufficient (Cronbach’s alpha .85 and .85).

### Procedure

Students completed the Beliefs about patient feedback questionnaire at the start of the clerkship (pre) and at the end of the facilitated reflection session (post). Students completed the Feedback perceptions questionnaire when they documented their feedback message in the online educational platform (pre) and at the end of the facilitated reflection session (post). Students filled in the questionnaires digitally. Completing the questionnaires was part of the regular curriculum. Due to logistical reasons, half of the students participated in the facilitated reflection session halfway through their clerkship (Group A), these students filled in the Feedback perceptions questionnaire for the feedback message they received from the gynecology patient. The other half of the students participated in the facilitated reflection session at the end of their clerkship (Group B), these students filled in the Feedback perceptions questionnaire for the feedback messages they received from the gynecology and the pediatric patient, see Figure 1.

**Figure 2:**
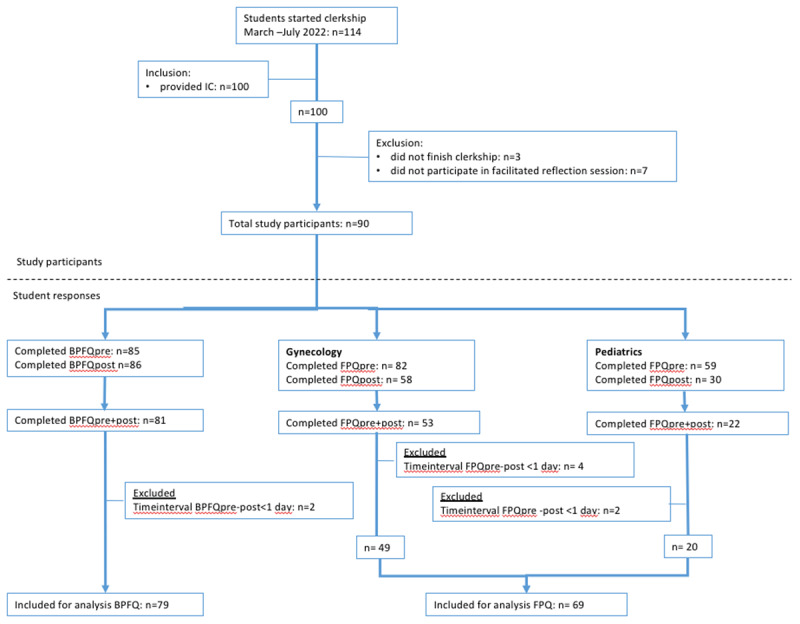
Flowchart of study participants and participant responses. BPFQ = beliefs about patient feedback questionnaire; FPQ = feedback perception questionnaire.

Ninety students were included in this study (see Figure 2). Students responses were paired through their student number and anonymized subsequently. In total, 37 students participated in the facilitated reflection session in week 7, and 53 participated in the facilitated reflection session in week 12. The Beliefs about patient feedback questionnaire pre and post was completed by 81 students. Two of these students were excluded for further analyses, because they had answered the first questionnaire only after already having had a feedback conversation with a patient, leaving 79 students for analysis. With regard to the Feedback perception questionnaire: 53 students completed the questionnaire pre and post for gynecology, four students were excluded because the time difference between pre and post was <1 day, leaving 49 students for analysis. For pediatrics, 22 students completed the feedback perception questionnaire pre and post. Two students were excluded because the time difference between the pre and post was <1 day, leaving 20 students for analysis.

For general beliefs about patient feedback we tested whether Beliefs about patient feedback scores were comparable between students who had their facilitated reflection sessions halfway or at the end of their clerkship. For feedback message perceptions we tested whether Perceived adequacy of feedback and Willingness to improve scores were comparable between messages from gynecology and pediatric patients. T-tests showed no significant differences (See Appendix 4). Therefore, in the analyses, all scores of the separate scales (BPF, FP_PAF_, FP_WI_) were considered as homogenous groups.

### Data analysis

To answer research question 1, we described mean scores of students’ Beliefs about patient feedback (BPF), students’ Perceived adequacy of the feedback message (FP_PAF_), and students’ Willingness to improve (FP_WI_). The Pearson’s correlation was performed to examine correlations between Perceived adequacy of the feedback and Willingness to improve.

To answer research question 2, about the differences before and after training in students’ general beliefs about patient feedback and feedback message perceptions, we performed paired t-tests for all three scales (BPF, FP_PAF_, FP_WI_). Effect size was measured with the Cohen’s D, which was calculated with the following formula: effect size = mean difference/standard deviation of the difference. To test whether changes in students’ general beliefs could be explained by students’ feedback message perceptions the Pearson correlation test was performed between the Beliefs about patient feedback and the Perceived adequacy of feedback.

## Results

### General beliefs about patient feedback and feedback message perceptions

At start of the clerkship, students’ mean scores regarding their Beliefs about patient feedback were 4.22 on a scale from 1–5 (SD = .45), with 93,7% of the students scoring 3.50 or higher. This indicates that most students had positive general beliefs about patient feedback. After training and experience, students’ mean scores regarding their Beliefs about patient feedback were 3.95 (SD = .53), with 88.6% of the students scoring 3.50 or higher (See Table 3; Appendix 5 shows the items table).

**Table 3:** Change in general beliefs and message perceptions of patient feedback after training.


	n	PRE MEAN (SD)	POST MEAN (SD)	Δ MEAN (SD)	df	p	d

Beliefs about patient feedback questionnaire							

BPF	79	4.22(.45)	3.95(.53)	–0.27 (.43)	78	<0.01	.63

Feedback message perceptions questionnaire							

FP_PAF_	69	7.97 (1.66)	7.32 (2.00)	0.65 (1.63)	68	<0.01	.40

FP_WI_	69	7.32 (2.42)	7.12 (2.34)	0.20 (1.78)	68	0.35	–


Δ = difference; n = number of students, SD = standard deviation, d = Cohen’s d, BPF = Beliefs about patient feedback, FPPAF = Perceived adequacy of feedback, FPWI = Willingness to improve.

Students’ initial mean scores of respectively the Perceived adequacy of the feedback and their Willingness to improve were 7.97 (SD = 1.66) and 7.32 (SD = 2.42) on a scale from 0-10. In total, 77.9% of the students scored a 7 or higher on Perceived adequacy of feedback and 59.4% scored a 7 or higher on Willingness to improve. This indicates that most students considered the feedback message adequate and were willing to improve. After the facilitated reflection session, students’ mean scores of respectively the Perceived adequacy of the feedback and their Willingness to improve were 7.32 (SD = 2.00) and 7.12 (SD = 2.34). In total, 62.3% of the students scored a 7 or higher on Perceived adequacy and 56.5% scored a 7 or higher on Willingness to improve, see Table 3. See Appendix 5 for the items table.

Correlation analysis showed a positive correlation between the Perceived adequacy of the feedback and the Willingness to improve, indicating that students who perceived higher adequacy of the feedback, reported to be more willing to use the feedback, see Table 4.

**Table 4:** Correlations between Perceived feedback adequacy and Willingness to improve.


	CORRELATION COEFFICIENT	p

Δ FP_PAF_ – Δ FP_WI_	.47*	<0.001

pre FP_PAF_ – pre FP_WI_	.50*	<0.01

post FP_PAF_ – post FP_WI_	.78*	<0.01


Δ = difference, FP_PAF_ = Perceived adequacy of feedback, FP_WI_ = Willingness to improve.

### Differences in general beliefs and feedback message perceptions before and after feedback training

Regarding students’ general beliefs, a paired t-test (assumptions met) showed that students’ beliefs about patient-feedback were significantly less positive after experience and training, see Table 3. The Cohen’s d was .63, which we consider as a moderate effect size.

Regarding students’ feedback message perceptions, a paired t-test (assumptions met) showed that after the facilitated reflection session students perceived the feedback message as less adequate. The Cohen’s d was .40, which we consider as a moderate effect size. There was no difference in student’s willingness to improve before and after the facilitated reflection, see Table 3.

We performed correlation analysis to explore whether the change in students’ beliefs about patient feedback could be related to their perception of the received feedback message or by the feedback training. Pearson correlation analyses showed no relevant correlation between the change in students’ Beliefs about patient feedback and their initial Perceived adequacy of the feedback message (r = .09, n = 74, p = .47), or the change in Perceived adequacy of the feedback message (r = .13, n = 48, p = .40).

## Discussion

This study explored (the development of) 4^th^-year medical students’ general beliefs about patient feedback and their specific perceptions of feedback messages during a clerkship by using adjusted validated questionnaires from peer-feedback literature. In this clerkship, students participated in patient feedback training and gained experience through feedback conversations with patients. Overall, students held positive perceptions about patients as feedback providers and about the feedback messages they received. However, students’ perceptions were less positive at the end of the clerkship, after training and experience in patient feedback. Below we will discuss these outcomes in light of literature and provide possible explanations for our results.

First, at the start of their clerkship, students had positive general beliefs about patient feedback. However, students’ perceptions were less positive after training and experience with patient feedback. Since receiving non-specific and mainly positive feedback from patients is often reported by (future) healthcare professionals, we explored whether the decline in positive beliefs could be explained by students being disappointed by the feedback message, as is previously seen in peer-feedback studies [22,28,32,39]. However, we found no correlations between the difference in students’ general beliefs and their specific perceptions of the adequacy of the feedback message, meaning students’ reported perceptions of the message did not explain the decline in their general beliefs about patient feedback.

Another possibility for the decline in positive perceptions about patient feedback could be that students realized it is difficult to obtain and engage with patient feedback. Kasch *et al*. reported that, in the context of peer-feedback during massive online open courses (MOOCs), students felt less prepared for peer-feedback after the course compared to before. They hypothesized this was due to students becoming more aware of the tasks related to receiving peer feedback [40]. The same could have happened in our population, students might have underestimated the complexity of their own active role in the process. This explanation is further supported by the fact that this was students’ first experience in patient feedback. After all, patient feedback was not part of the formal curriculum in other clerkships at the time of the study. It is known from peer-feedback studies that students without experience are more positive at start than students with some experiences in peer-feedback [40].

Secondly, most students perceived the received feedback message as adequate (respectively 78% and 60%, before and after facilitated reflection) and were willing to improve (respectively 62% and 57% before and after facilitated reflection). This exceeds the results of the questionnaire study of Gran *et al*., where out of 79 students only half of the students considered the patient feedback they received as useful [27]. Studies in other contexts, psychology or secondary school students receiving peer feedback on writing tasks, also report lower means of perceived adequacy of feedback and willingness to improve than the means we found in this study [31,41,42]. This shows that the medical students from our study hold at least promising perceptions towards patient feedback messages, compared to findings with the same instruments in other contexts of feedback. However, students perceived the feedback message as less adequate after participating in a facilitated reflection session focused on sense-making and action-planning. Interestingly, other studies found that for doctors facilitated reflections sessions did benefit the acceptance and interpretation of patient feedback and subsequent goal-setting [4,22]. Possibly, the decline can be explained by the instructional design of the facilitated reflection session. Room for emotions and reflective discussions are identified as important elements of facilitated reflection to support feedback acceptance and use [4]. Even though, these aspects were part of the instructional design, perhaps reflection still was not reached.

Another possibility for the decline in positive feedback message perception, is that students obtained mainly positive, unspecific feedback, as is often reported in patient feedback literature [22,28,32,43]. Students might have initially been content with this feedback, as positive feedback is more often congruent with self-perceptions and therefore more likely to be accepted [4,44]. Yet, during facilitated reflection, students might became aware that constructive specific feedback would have been more helpful to develop new learning goals.

Our results indicate that patient feedback is not an easy means to learn and students do not become feedback literate in terms of patient feedback overnight [45]. Therefore, we suggest implementing longitudinal feedback training, where appreciation for patient feedback is fostered and students are guided in asking patients for informative feedback, in sense-making of received feedback and in subsequent action-planning [46]. Longitudinal training can support students in overcoming common barriers and pitfalls [45,47]. Ideally, patient feedback training is integrated in a longitudinal program focused on supporting student feedback literacy, where students learn to integrate feedback from various sources [1,45,48]. Moreover, mentoring at the workplace can support students in feedback engagement, which requires teaching the teachers on how to support students in engaging with patient feedback [49,50].

This study has some limitations. Our study showed a decrease in students’ positive general beliefs about patient feedback and feedback message perceptions after training and experience. However, the available data did not allow explanation of these declines. Understanding the reasons for these declines could help reverse or prevent them and thereby support engagement with patient feedback. Unfortunately, the ethical approval for the study led to anonymizing the data. Therefore, we could not approach specific students for follow-up interviews to gain a deeper understanding of the current findings. We suggest for future research to combine questionnaire data with qualitative methods like interviewing to provide explanations for changes in students’ perceptions. Furthermore, to understand the role of the feedback message, we suggest for future research to collect actual feedback messages and compare aspects of these messages (e.g. specific vs. non-specific, positive vs. critical) with students’ beliefs and perceptions. Our study was designed to have a rather short follow-up. It remains unknown how beliefs about patient feedback further develop throughout medical school and beyond. Peer-feedback research shows an initial decline of positive peer-feedback perceptions, which is then followed by an overall increase [40]. Possibly beliefs about patient feedback show a similar pattern. Future research could employ longitudinal study designs following students throughout their education, or cross-sectional study designs comparing beliefs of students and healthcare professionals in various phases of their education or career. The two questionnaires we developed in this study by building on peer feedback research, could be employed by future studies.

In conclusion, students’ beliefs about patient feedback had been reported in the literature to be positive, but had often been measured at only a single point in time [26,27,28]. Our study showed that students’ beliefs about patient feedback become less positive after initial training and experience. Moreover, we found a downward shift in students’ positive perceptions of patients’ feedback messages after facilitated reflection. It seems that students start with high hopes and enthusiasm about patient feedback, but lose some of this positivity when they actively engage with patient feedback. Clearly, (patient) feedback is not an easy means to learn [47] and requires more research on how to come to full potential. Specifically, future research would need to explore the reasons for the decline in students’ positive beliefs and perceptions, and explore ways to support students (and patients) in the complex task of learning from patients through feedback.

## Additional File

The additional file for this article can be found as follows:

10.5334/pme.1261.s1 Supplementary File. Appendix 1 to 5.
