# Supplementary material for: Medical Students’ General Beliefs and Specific Perceptions about Patient Feedback Before and after Training in a Clinical Context
Source: Perspect Med Educ. 2024 May 8;13(1):288–99. doi: 10.5334/pme.1261 (PMC11086596; doi:10.5334/pme.1261)
Supplement: Supplementary File. — Appendix 1 to 5. [file pme-13-1-1261-s1.pdf]

APPENDIX 1 – Set-up of Facilitated reflection session

Duration: 1 hour, 45 minutes
Participants: 10-15 students

Time schedule

0-5 min	Introduction
5-25 min	Reflection on feedback conversation
25-40 min	Emotional impact
40-70 min	Sense-making feedback message
70-95 min	Action-planning
95-105 min	Closure

Reflection on feedback conversation

Students reflect on their feedback conversations and compare these to 4 criteria of the Westerveld framework for feedback dialogues: open & respectful, relevant, timely, dialogical.(ref)
Using Wooclap and plenary discussion we discuss the following questions:

- Which criteria did they meet? How did this contribute to their feedback dialogue?
- Which criteria did they not meet? Why not?
- What would you do the same next time? What would you do differently?

Emotional impact

The emotional impact of feedback is discussed.
By diving students in duo's, followed by plenary discussion we discuss the following questions:

- How did you feel after receiving patient feedback? Why? How did you handle this emotional impact?
- Did you experience negative emotions? Which ones? Why? How did you handle this emotional impact?

When a student experienced negative emotions, the option to discuss this after the session one-on-one with the teacher is provided.

Sense-making feedback message

Students analyze and interpret their feedback message(s) by making comparisons.(ref Nicol)
First, the feedback message of one student is analyzed plenary, so students become familiar with the comparisons. Then students are given a hand-out: they fill in the first part individually, followed by group discussion. Students answer the following questions by filling in the hand-out:

- What was the feedback message?
- How does the feedback message compare to my self-assessment of the situation?
- How does the feedback message compare to your learning goal and criteria for this goal?
- How does the feedback message compare to feedback from others and previous situations?
- What is your conclusion regarding the feedback? (accept/reject)
- What is your conclusion regarding your learning goal?

Action-planning

Students develop an action plan to use the feedback they received.

First, students individually fill in the second part of the hand-out where they answer the questions below. Second, they discuss their action plan in duo's. Lastly, action plans are discussed in plenary discussion.

- Based on your conclusions regarding the feedback and your learning goal, what did you learn about your strengths and weaknesses of your performance?
- How are you going to implement what you learned in future situations? When, where, and how can you apply what you learned?
- What will be your new learning goal? Or: how will you adjust your previous learning goal?
- How are you going to reach this (adjusted) learning goal?

Hand-out sense-making and actionplanning patient feedback

Feedback message	**What was the content of the feedback messages you received?**<br>Patient 1 (gynecology):<br><br>Patient 2 (pediatrics):
Sense-making	**How does the feedback message compares to:**<br>- your self-assesment of the situation<br>- your learning goal and criteria for this goal<br>- feedback from others and previous situations<br><br>What is your conclusion regarding the feedback?<br><br>What is your conclusion regarding your learning goal?
Action-planning	**Taking action**<br>- What did you learn about your strengths and weaknesses of your performance?<br><br>- How are you going to implement what you learned in future situations? (specify when, where, and how you will apply what you learned)<br><br>- What will be your new learning goal / how will you adjust your previous learning goal?<br><br>- How are you going to reach this (adjusted) learning goal?

APPENDIX 2 - Factor loadings BPFQ

Table: PCA loadings (n=85) of Beliefs about Patient Feedback Questionnaire (BPFQ) with oblique (oblimin) rotations, non-fixed

Scale	Items	Component I
**VIM**	VIM1	0.81
	VIM2	0.74
	VIM3*	0.60
**CR**	CR1	0.51
	CR2	0.70
**VPS**	VPS1	0.63
	VPS2	0.66
**Eigenvalues**		3.14
**% of variance explained**		44.78

*reverse

APPENDIX 3 - FPQ principal component analysis

The FPQ consists of 5 scales, namely fairness (FA, 3 items), usefulness (US, 3 items), acceptance (AC, 3 items), willingness to improve (WI, 3 items) and affect (6 items). In our study we used 4 out of 5 scales: FA, US, AC, WI. The FA, US and AC together constitute a second order scale, namely the perceived adequacy of feedback (PAF). The PAF relates to the cognitive function of feedback. The WI relates to the motivations function of feedback.
The underlying structure of our adjusted questionnaire was explored by conducting factor analyses on the FPQ data of the first moment students filled in this questionnaire. The pattern matrix and scree plot were used to determine the number of components, and factor loadings were used to interpret the components. First, we performed a non-fixed principal component analysis with direct oblimin rotation. This resulted in four components, which together explained for 75,80% of the total variance, see the table 1 below for factor loadings. However, the structure of the questionnaire did not follow the original structure (FA, US, AC and WI) as described by Strijbos *et al.,* which would hinder comparability of our results with other studies.[29] Therefore, we perfomed a second analysis: we tested whether the questionnaire could be divided in a cognitive and motivational dimension. We performed a principal component analysis with the factor amount fixed on 2. This resulted in two components that explained for 57,06% of the total variance, and which matched the original structure: component 1 consisted of the fairness, usefulness and acceptance items, reflecting the cognitive function in term of the PAF scale; component 2 consisted of the willingness to improve items, reflecting the motivational function. Thus, we decided to use the PAF and WI scale for analyses. Factor loadings for the two scales ranged from 0,46-0,91, see table 2 below. The internal consistencies of the PAF and WI scale had sufficient reliability (Cronbach's alpha>0.8).

Table 1: PCA loadings (n=82) of Feedback Perception Questionnaire with oblique (oblimin) rotations, non-fixed

		**Components**			
**Scale**	**Items**	**I**	**II**	**III**	**IV**
**Fairness**	FA1	0.40	-1,.22	**-0.65**	-0.77
	FA2	-0.17	0.15	**-0.75**	0.24
	FA3	0.12	0.13	**-0.79**	-0.0060
**Usefulness**	US1	**0.91**	0.015	0.0040	0.081
	US2	**0.71**	0.12	-0.26	0.13
	US3	**0.81**	0.0090	-0.084	0.042
**Acceptance**	AC1	0.093	-0.050	**-0.85**	-0.072
	AC2*	-0.074	-0.072	-0.091	**0.87**
	AC3*	0.20	0.014	0.093	**0.72**
**Willingness to improve**	WI1	0.25	**0.84**	0.15	-0.025
	WI2	-0.079	**0.90**	-0.083	0.013
	WI3	-0.12	**0.87**	-0.14	-0.048
**Eigenvalues**		4.68	2.17	1.17	1.080
**% of variance explained**		38.97	18.10	9.74	9.00

*reversed

Table 2: PCA loadings (n=82) of Feedback Perception Questionnaire with oblique (oblimin) rotations, fixed on two factors

		**Components**	
**Scale**	**Items**	**I**	**II**
**Fairness**	FA1	**0.78**	0.042
	FA2	**0.52**	0.33
	FA3	**0.67**	0.33
**Usefulness**	US1	**0.81**	-0.067
	US2	**0.85**	0.11
	US3	**0.77**	-0.035
**Acceptance**	AC1	**0.66**	0.19
	AC2*	**0.46**	-0.17
	AC3*	**0.49**	-0.14
**Willingness to improve**	WI1	0.065	**0.76**
	WI2	-0.026	**0.91**
	WI3	-0.056	**0.90**
**Eigenvalues**		4.68	2.17
**% of variance explained**		38.97	18.10

*reversed

APPENDIX 4 - Correlations between groups

<u>BPFQ group A and B</u>

t-test

	**Group A**	**Group B**	**Difference**	**df**	**p**
BPFQpre mean (SD)	4.22(0.52)	4.23 (0.39)	0.010 (0.10)	77	0.92
BPFQpost mean (SD)	3.96 (0.53)	3.94 (0.54)	-0.023 (0.12)	77	0.85
ΔBPFQ mean (SD)	-0.25 (0.43)	-0.28 (0.45)	0.026 (0.099)	77	0.89

<u>FPQ questionnaire gynecology and pediatrics</u>

t-test

	**Gynecology**	**Pediatrics**	**Difference**	**df**	**p**
PAFpre (SD)	8.08 (1.30)	7.88 (1.81)	0.19 (0.26)	138	0.46
PAFpost (SD)	7.29 (1.74)	7.55 (2.05)	0.26 (0.42)	86	0.53
WIpre (SD)	7.25 (2.23)	7.45 (2.01)	0.20 (0.36)	138	0.58
WIpost (SD)	7.36 (2.15)	6.98 (2.31)	0.38 (0.50)	86	0.44

APPENDIX 5 – Item tables BPFQ and FPQ

Item table BPFQ

	Pre					Post				
	n.	mean	SD	min	max	n.	mean	SD	min	max
Valuation of patient-feedback as an instructional method										
VIM1	79	4.37	0.68	2	5	79	4.01	0.73	2	5
VIM2	79	4.20	0.72	2	5	79	3.57	0.89	1	5
VIM3	79	1.68	0.79	1	4	79	1.75	0.81	1	4
Confidence in quality of received patient-feedback										
CR1	79	3.72	0.58	2	5	79	3.20	0.74	1	5
CR2	79	4.09	0.64	2	5	79	3.70	0.81	2	5
Valuation of peer-feedback as an important skill										
VPS1	79	4.48	0.57	3	5	79	4.56	0.64	3	5
VPS2	79	4.39	0.63	2	5	79	4.35	0.64	2	5

Item table FPQ

	Pre					Post				
	n.	mean	SD	min	max	n.	mean	SD	min	max
Fairness										
FA1	69	8.23	2.07	0	10	69	7.23	2.67	0	10
FA2	69	8.42	1.87	1	10	69	7.81	2.32	1	10
FA3	69	8.32	1.71	2	10	69	8.01	2.37	0	10
Usefulness										
US1	69	7.28	2.39	1	10	69	6.39	2.81	0	10
US2	69	7.38	2.24	1	10	69	6.45	2.63	0	10
US3	69	6.58	2.62	0	10	69	5.52	2.52	0	10
Acceptance										
AC1	69	8.77	1.80	1	10	69	8.57	2.25	0	10
AC2	69	1.38	2.34	0	10	69	1.86	2.66	0	10
AC3	69	1.69	2.75	0	10	69	1.91	2.67	0	10
Willingness to improve										
WI1	69	6.99	2.91	0	10	69	7.29	2.50	0	10
WI2	69	7.01	2.73	0	10	69	6.74	2.42	0	10
WI3	69	7.97	2.19	0	10	69	7.33	2.61	0	10

N= number of students; SD = standard deviation

FA1 = I am satisfied with this feedback
FA2 = I consider this feedback fair
FA3 = I consider this feedback justified
US1 = I consider this feedback useful
US2 = I consider this feedback helpful
US3 = This feedback provides me a lot of support
AC1 = I accept this feedback
AC2 = I dispute this feedback
AC3 = I reject this feedback
WI1 = Based on this feedback, I am willing to improve my performance
WI2 = I am willing to invest a lot of effort in improving the skills I received feedback on
WI3 = I am willing to, during my clerkships, work on further developing the skills I received feedback on
